# Supplementary material for: Extracellular Matrix and Fibrocyte Accumulation in BALB/c Mouse Lung upon Transient Overexpression of Oncostatin M
Source: Cells. 2019 Feb 5;8(2):126. doi: 10.3390/cells8020126 (PMC6406700; doi:10.3390/cells8020126)
Supplement: Supplementary file 1 [file cells-08-00126-s001.pdf]

Supplementary Figure 1

A

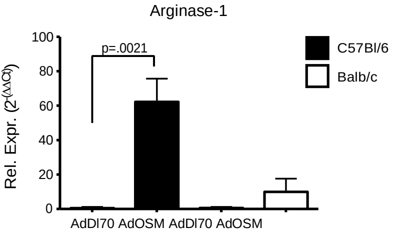

B

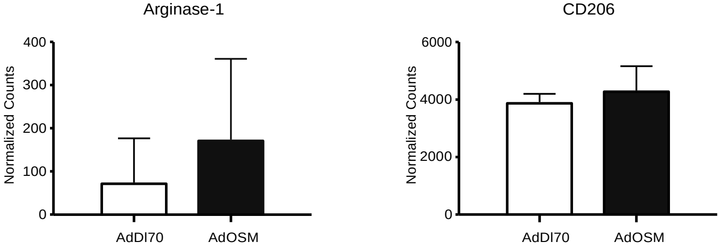

Supplementary Figure 1. Expression of Arginase-1 in mouse whole lung and Arginase-1 and CD206 in alveolar macrophages. (**A**) Wildtype C57Bl/6 or BALB/c mice were endotracheally administered AdDl70 or AdOSM (5×10^7 pfu), culled after 7 days and lung tissues isolated and prepared for RNA extraction. RNA was probed for Arginase-1 by quantitative PCR. (**B**) Alveolar macrophages were isolated, as previously described (49), from the lungs of BALB/c mice treated with AdDl70 or AdOSM (as described in (**A**)) and RNA extracted and probed for expression of Arginase-1 and CD206 was assayed using Nanostring technology (Nanostring Technologies, Seattle, WA). Data is shown as the mean +/- SEM ($n = 5$ per group). Statistical significant differences are noted with their p values between the indicated treatment groups.
